# Spontaneous Closure of Bronchopleural Fistula after Right Upper Lobectomy without Invasive Interventions—A Case Report

**DOI:** 10.70352/scrj.cr.25-0432

**Published:** 2025-12-04

**Authors:** Taketo Kato, Heng Huang, Taiki Ryo, Yoshito Imamura, Yuji Nomata, Hirofumi Takenaka, Hiroki Watanabe, Yuta Kawasumi, Keita Nakanishi, Yuka Kadomatsu, Harushi Ueno, Shota Nakamura, Tetsuya Mizuno, Toyofumi Fengshi Chen-Yoshikawa

**Affiliations:** Department of Thoracic Surgery, Nagoya University Graduate School of Medicine, Nagoya, Aichi, Japan

**Keywords:** bronchopleural fistula, spontaneous closure, diabetes mellitus, lung cancer, surgery

## Abstract

**INTRODUCTION:**

A bronchopleural fistula (BPF) is a serious and potentially life-threatening complication of pulmonary resection, with a particularly high incidence following pneumonectomy. Although surgical repair is the mainstay of treatment, conservative management with bronchoscopic intervention also results in complete resolution in some cases. Spontaneous closure of the BPF, especially after full dehiscence of the bronchial stump, remains exceptionally uncommon.

**CASE PRESENTATION:**

A 60-year-old man with diabetes mellitus and interstitial pneumonia underwent right upper lobectomy for suspected lung cancer. The tumor adhered to the superior vena cava, requiring pericardial dissection and phrenic nerve resection. The bronchial stump was sutured and covered with a free pericardial fat pad. Postoperative recovery was initially uneventful; however, on POD 18, the patient presented with dyspnea and was diagnosed with right lower lobe pneumonia. On POD 31, he spat out all suture material and fat tissue with hemosputum. Imaging findings confirmed the presence of a BPF at the bronchial stump without pneumothorax or empyema. He was then conservatively managed with antibiotics and glycemic control. The hilar air space gradually decreased over the following weeks, and CT confirmed complete spontaneous closure of the fistula by POD 151.

**CONCLUSIONS:**

Our case highlights that, under selected conditions—such as absence of empyema, confined necrosis of the bronchial stump, and reduced residual pleural space due to phrenic nerve paralysis and adhesions surrounding the hilum—spontaneous closure of a BPF without a surgical or bronchoscopic intervention is possible. Conservative management with careful monitoring may be a feasible option in selected patients.

## Abbreviations

BPF
: bronchopleural fistula
CRP
: C-reactive protein
SVC
: superior vena cava
WBC
: white blood cell

## INTRODUCTION

A BPF is a pathological communication between the bronchial tree and pleural space, representing one of the most serious and life-threatening complications following lung resection surgery, with reported mortality rates ranging from 16% to 72% depending on the clinical scenario and timing of onset.^1)^ The incidence of BPF varies by surgical procedure, occurring in approximately 0.5%–1% and 4.5%–20% of patients undergoing lobectomy and pneumonectomy, respectively.^2)^ The development of BPF may lead to catastrophic consequences, including tension pneumothorax, empyema, aspiration pneumonia, and respiratory failure, necessitating prompt diagnosis and appropriate management.

Although no standardized therapeutic approach has been established yet, surgical repair, including open window thoracostomy, reportedly can achieve a success rate of 75%–100% in selected patients.^3)^ Bronchoscopic approaches have recently evolved from purely diagnostic tools to valuable therapeutic alternatives, particularly in patients deemed unsuitable for surgery. However, few cases have successfully achieved a cure through nonsurgical technique and conservative treatment. Herein, we present a rare case of spontaneous closure of a BPF after right upper lobectomy without any surgical or bronchoscopic intervention.

## CASE PRESENTATION

**Fig. 1:**
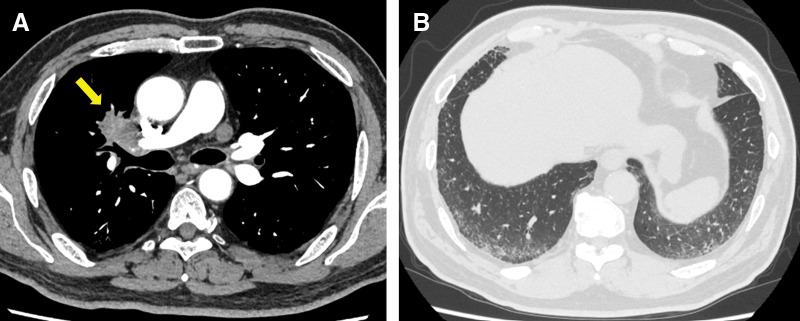
Preoperative CT findings. (**A**) Contrast-enhanced CT revealing a tumor measuring 5.0 cm in diameter in the right upper hilum (yellow arrow). (**B**) Interstitial pneumonia was observed at the bases of the bilateral lungs.

A 60-year-old man was referred to our hospital for surgery of a tumor in the right lung, which was detected on CT by his previous physician. Chest CT showed a 5.0-cm tumor in the hilum of the right upper lung, complicated by interstitial pneumonia in both lower lobes (**Fig. 1A** and **1B**). Although no definite diagnosis was obtained by transbronchial lung biopsy, lung cancer was strongly suspected. Fluorodeoxyglucose-PET showed a high accumulation in the tumor (maximum standardized uptake was 24.0). The right hilar node showed an accumulation, but no mediastinal nodes or distant metastases were noted. He had a history of diabetes mellitus with a hemoglobin A1c level of 8.4%. Therefore, a right upper lobectomy was planned after 1 week of hospitalization for glycemic control.

Surgery was performed via thoracotomy with the patient under general anesthesia. The tumor was found in the right upper lobe and had adhered to the SVC. We opened the pericardial sac and secured the SVC. Although phrenic nerve resection was necessary, the tumor was removed from the SVC by careful dissection. After resecting the pulmonary vein and artery of the right upper lobe with staplers, the tumor was dissected from the right upper bronchus, and no thermal injury to the bronchus was noted. Intraoperative pathological examination revealed no malignancy at the bronchial stump; hence, closure with 3-0 polypropylene suturing was performed. The upper mediastinal and subcarinal lymph nodes were also dissected, and a free pericardial fat pad was used to cover the bronchial stump. The patient’s postoperative course was uneventful. The chest tube was removed on POD 2, and the patient was discharged on POD 7 without complications. The final pathological diagnosis was adenosquamous carcinoma, classified as pT2aN0M0, pStage IB.

**Fig. 2:**
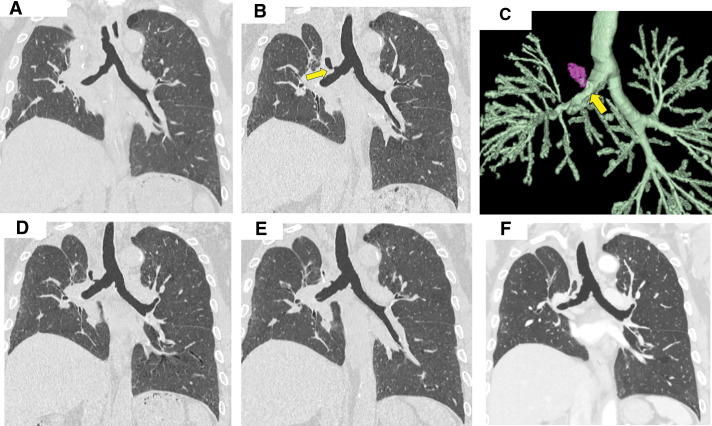
Postoperative CT scans obtained before and after the diagnosis of BPF. (**A**) CT scan on POD 18 showed pneumonia in the right S6 segment, without any abnormal findings at the bronchial stump. (**B**) CT scan on POD 32. The right upper bronchial stump communicated with a cystic structure at the right bronchial hilum (yellow arrow). (**C**) 3D CT on POD 32 demonstrating direct communication between the bronchial stump (yellow arrow) and the hilar cystic cavity (purple area). (**D**) CT scan on POD 55 showed a reduction in the size of the cystic lesion. (**E**) On POD 69, the BPF was almost closed. (**F**) CT scan on POD 206 demonstrated complete closure of the BPF without evidence of bronchial stenosis or residual air space. BPF, bronchopleural fistula

**Fig. 3:**
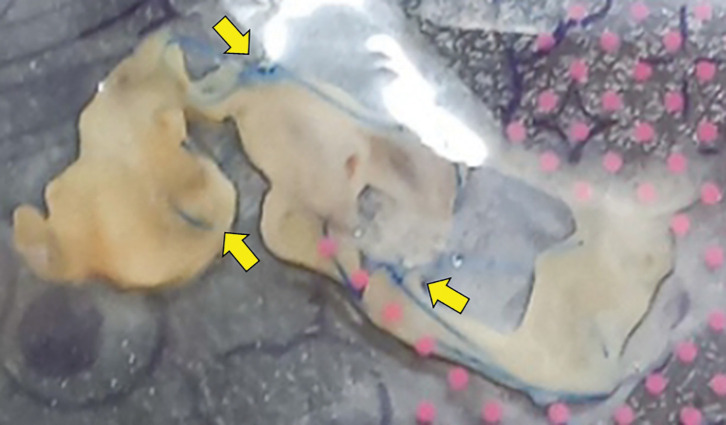
Suturing material (yellow arrow) and pericardial fat tissue coughed up by the patient.

**Fig. 4:**
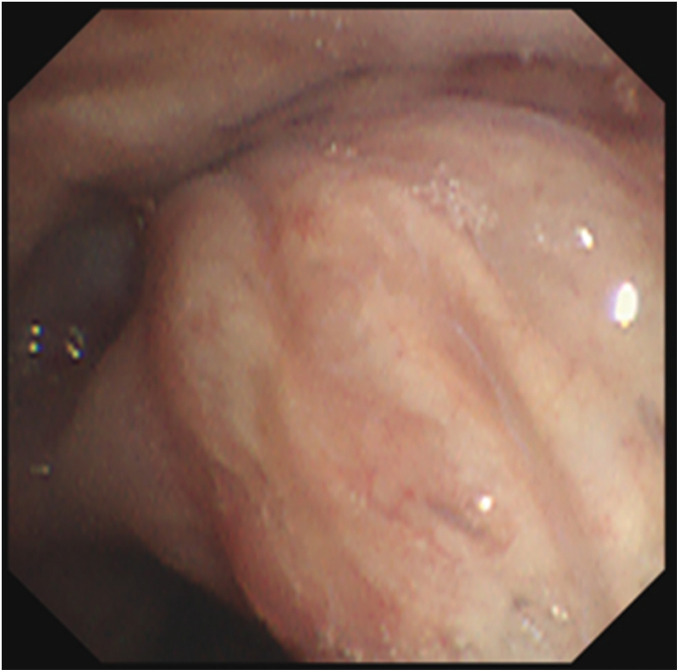
Bronchoscopy findings at 60 days after surgery. There was no sign of necrosis in the bronchial mucosa, and the bronchopleural fistula had nearly closed.

The patient presented with dyspnea at our hospital on POD 18. His body temperature was 37.0°C, SpO_2_ was 96% on room air, WBC was 12200/µL, and CRP was 8.81 mg/dL. Chest CT revealed pneumonia in the right S6 segment and a limited air space around the right hilum, without any communication with the bronchial stump (**Fig. 2A**). The pneumonia was considered bacterial in nature, as KL-6 levels were not elevated and no abnormal findings were observed at the bronchial stump on CT. Oral antibiotic therapy was initiated; however, he reported expectorating pericardial fat tissue and a suturing string with hemosputum on POD 31 (**Fig. 3**). His body temperature was 36.6°C, SpO_2_ was 96% on room air, WBC was 8700/µL, and CRP was 0.42 mg/dL. Follow-up chest CT on POD 32 showed a communication between the bronchial stump and the air space in the right hilum, without evidence of pneumothorax or empyema (**Fig. 2B**). 3D CT imaging obtained at that time demonstrated direct communication between the bronchial stump and the hilar cystic cavity (**Fig. 2C**). After spitting out the suture, the inflammatory markers, including WBC count and CRP levels, were not elevated. Based on these findings, the patient was diagnosed with BPF without an associated infection. We opted to perform conservative management with prophylactic oral antibiotics and glycemic control during hospitalization. The patient did not develop any infection during the 1-month hospital stay, and serial chest CT scans showed gradual reduction of the hilar air space (**Fig. 2D**). Bronchoscopy confirmed the bronchial stump’s integrity with no signs of necrosis (**Fig. 4**). The patient was then discharged on POD 70, and complete spontaneous closure of the BPF was confirmed by chest CT on POD 151 (**Fig. 2E**). The most recent CT obtained on POD 206 demonstrated complete closure of the BPF without evidence of bronchial stenosis or residual air space (**Fig. 2F**).

## DISCUSSION

BPF remains one of the most challenging complications following pulmonary resection and is traditionally associated with high morbidity and mortality rates.^4)^ Although surgical interventions, including open window thoracostomy and muscle flap coverage, are often required in severe cases,^5)^ accumulating evidence suggests that conservative management may be effective in selected patients. Several studies have demonstrated the efficacy of conservative approaches comprising appropriate pleural drainage, targeted antibiotic therapy, and bronchoscopic interventions.^6,7)^ However, spontaneous closure of a BPF without drainage and bronchoscopic interventions is rare, with only limited cases previously reported in the literature.^8)^ In particular, reports detailing the process of spontaneous closure of a BPF after complete dehiscence of the right upper bronchial stump without any invasive treatment are lacking.

The development of BPF is often multifactorial and patient-specific. Several potential risk factors, including both local and systemic contributors, have been implicated. One of the most frequently identified systemic risk factors is diabetes mellitus, which impairs wound healing and is associated with delayed bronchial stump repair. As an operative procedure, right pneumonectomy or right lower lobectomy is a recognized risk factor. Local ischemia due to excessive dissection or devascularization of the bronchial stump during hilar lymphadenectomy is another key factor, as this can lead to bronchial wall necrosis, which compromises the closure integrity. Additionally, postoperative pulmonary infections play a considerable role in disrupting bronchial healing, especially in the early postoperative period.^9)^ In the present case, local ischemia due to abrupt dissection around the bronchial stump, particularly during manipulation near the pulmonary hilum and SVC, is suspected. Additional contributing factors include underlying diabetes mellitus and postoperative pneumonia.

As a factor of spontaneous BPF closure, conservative management may be considered appropriate under the following conditions: (1) re-expansion of the residual lung is expected; (2) necrosis or ischemia is confined to the bronchial stump; and (3) aspiration pneumonia in the contralateral lung is absent or minimal.^10)^ Although conditions (2) and (3) were true in the present case, the preoperative volume of the right upper lobe, which contained the tumor, was larger than that of the right lower lobe due to interstitial pneumonia (the right upper and lower lobe volumes were 40.4% and 37.2%, respectively, of the total right lung volume). Additionally, the residual right middle and lower lobes were not expected to expand well due to fibrosis. Therefore, other factors should be considered for achieving BPF closure with conservative management without bronchial fiber intervention or tube drainage. A possible mechanism in this case involves containment of the infection within a localized pleural space, supported by tight pleural adhesions around the fistula. Because phrenic nerve paralysis and aggressive hilar dissection resulted in a markedly small residual thoracic cavity from an early stage, the BPF was likely predisposed to remain confined rather than to develop into a diffuse empyema. In addition, the patient’s infectious and general condition was well-controlled throughout the clinical course. Together, these factors may have contributed to the spontaneous closure of the BPF without the need for invasive intervention. In this case, one limitation is that the precise extent and location of necrosis or ischemia were not confirmed bronchoscopically at the time when the fat pad was expectorated. However, the 3D CT findings demonstrated direct communication between the bronchial stump and the cystic hilar cavity, suggesting that the fistula originated at the bronchial stump rather than resulting from bronchial injury caused by manipulation during lymph node dissection.

Even when conservative management appears appropriate, careful and continuous follow-up is essential to monitor for progressive enlargement of the fistula or expansion of the empyema cavity. If clinical deterioration occurs, surgical interventions—such as open-window thoracostomy or completion pneumonectomy—should be reconsidered, particularly in emergent situations such as hemoptysis due to pulmonary artery involvement. In the present case, prophylactic resection of the BPF, including right pneumonectomy or bronchial sleeve resection, was not performed due to the high risk of postoperative respiratory failure, acute exacerbation of interstitial pneumonia, and the possibility of BPF recurrence following such surgical procedures.

## CONCLUSIONS

Although exceedingly rare, spontaneous BPF closure without an invasive intervention is possible under selected conditions, such as the absence of empyema, confined necrosis of the bronchial stump, and reduced residual pleural space due to phrenic nerve paralysis and adhesions surrounding the hilum. Precise patient selection, early detection, and radiologic and bronchoscopic surveillance are essential to ensure successful conservative management of patients.

## DECLARATIONS

### Availability of data and materials

Data sharing is not applicable to this article as no datasets were generated or analyzed during the current study.
